# Increased circulating innate lymphoid cell (ILC)1 and decreased circulating ILC3 are involved in the pathogenesis of Henoch-Schonlein purpura

**DOI:** 10.1186/s12887-022-03262-w

**Published:** 2022-04-12

**Authors:** Lili Zhang, Qiang Lin, Lijun Jiang, Mingfu Wu, Linlin Huang, Wei Quan, Xiaozhong Li

**Affiliations:** 1 Department of Nephrology, Children’s Hospital of Soochow University, No. 92, Zhong Nan Street, Industrial Park, Suzhou, 215003 Jiangsu China; 2 Department of Pediatrics, Affiliated Hospital of Yangzhou University, Yangzhou, 225000 Jiangsu China; 3 Department of Neonatology, Affiliated Hospital of Yangzhou University, Yangzhou, 225000 Jiangsu China; 4 Department of Pediatric Intensive Care Unit, Children’s Hospital of Soochow University, Suzhou, 215003 Jiangsu China

**Keywords:** Henoch-Schonlein purpura, Innate lymphoid cells, Lymphocyte subpopulation, Lymphocytes

## Abstract

**Background:**

Innate lymphoid cell (ILC) dysfunction is involved in numerous immune diseases, but this has not been demonstrated in Henoch-Schonlein purpura (HSP). This study aimed to investigate whether ILC dysfunction or imbalance participate in the pathogenesis of HSP.

**Methods:**

This was a prospective study in patients with HSP who were hospitalized at the Children’s Hospital of Soochow University from June to December 2019. Age- and sex-matched controls were also enrolled. ILC subsets and lymphocyte subpopulations were determined by flow cytometry. The transmission immune turbidimetric method also facilitated the exploration of correlations between ILC subset frequency and lymphocyte subpopulation, as well as serum IgA in HSP patients.

**Results:**

Fifty-one patients with HSP and 22 control patients were included. There were no differences in age and sex between the two groups. Compared with controls, patients with HSP had higher ILCs in relation to lymphocytes (*P* = 0.036), higher ILCs in relation to PBMCs (*P* = 0.026), higher ILC1s (*P* < 0.001), lower ILC3s (*P* < 0.05), and higher ILC1/ILC3 ratio (*P* < 0.001). Sixteen patients underwent routine therapy combined with methylprednisolone for 7–10 days; ILC1s were significantly decreased (*P* < 0.001) and ILC3s were increased (*P* = 0.033), and ILC1/ILC3 was significantly decreased (*P* < 0.001). Compared with the controls, the ratios of ILCs/lymphocytes and ILCs/PBMC were higher in patients in the arthritis and mixed groups (all *P* < 0.05). ILC1 were elevated in the purpura, arthritis, abdominal, and mixed groups (*P* = 0.027, *P* = 0.007, *P* < 0.001, and *P* < 0.001, respectively). ILC1/ILCs were positively correlated with CD3 + CD8 + T lymphocytes (*r* = 0.3701, *P* = 0.0075). The level of IgA did not correlate with ILCs.

**Conclusions:**

Higher circulating ILC1s and lower circulating ILC3s appear to be involved in the pathogenesis of HSP.

**Supplementary Information:**

The online version contains supplementary material available at 10.1186/s12887-022-03262-w.

## Background

Henoch-Schonlein purpura (HSP), also known as IgA vasculitis, is an acute systemic immune-mediated small vessel vasculitis resulting in palpable purpura without thrombocytopenia, variable manifestations of abdominal pain, arthritis or arthralgia, proteinuria, hematuria, and IgA deposits in small vessels (which may be demonstrated on the skin or kidney biopsy) [1–4]. HSP occurs mainly in children < 10 years old but may affect all ages [2, 5]. It is relatively uncommon (the highest reported incidence at a peak age of 4–6 years is 0.07%) [1, 5]. HSP is usually self-limited with spontaneous resolution within 4 weeks in 94% of children and 89% of adults, but with a 30%-40% recurrence rate within the first year [1, 2, 6]. HSP nephritis (HSPN) is the most serious complication. The long-term prognosis of HSPN includes chronic kidney disease, which accounts for 1%-2% of end-stage renal diseases [1, 2, 7].

HSP involves the formation of IgA complexes that deposit into the skin, gastrointestinal tract, and kidney and will result in small blood vessel necrosis [1, 2]. Besides IgA, the other players in HSP include IgG, IgM, T lymphocytes, as well as other immune cells, cytokines, and complement [8]. The damaged epithelial cells may secrete cytokines and promote the activation and proliferation of innate lymphoid cell (ILCs) [9, 10].

The ILC family is a group of innate effector cells that are enriched in the barrier surfaces and provides early and prompt defensive immune response to protect epithelial integrity and tissue immunity [11]. ILCs are characterized by the lack of antigen specificity for T and B cells or myeloid and dendritic cell phenotypical markers [12]. According to their origin and function, ILCs are divided into five subsets, type 1, 2, and 3 ILC subsets, natural killer (NK) cells, and lymphoid-tissue inducer (LTi) cells [13]. ILCs exhibit a trait of functional diversity and plasticity. ILC2 and ILC3 can be converted into ILC1 in the presence of IL-12, IL-18, and IL-1β, and conversely, ILC1 can be converted back to ILC2 and ILC3 in the presence of IL-4 or IL-23, respectively [14, 15]. Therefore, different immune diseases or conditions characterized by different cytokine profiles might shift the balance between ILC1 and ILC2/3 [14, 15].

ILCs are important regulators of the epithelial barrier and are involved in immune defense. ILCs are involved in the pathogenesis of a range of chronic infectious, inflammatory, or metabolic diseases in humans, and they play an important role in maintaining mucosal barriers and tissue repair and removing parasitic infections and a variety of tumors [16]. ILC2 plays a pathogenic role in pulmonary allergy and inflammatory processes [17–20]. ILC2 induces allergic pulmonary inflammation by secreting IL-13, which promotes the migration of activated DCs to draining lymph nodes and initiates Th2 differentiation [21]. IL-22 produced by ILC3 is also associated with asthma pathogenesis due to the elevated levels of IL-22 in asthmatic patients compared to healthy controls [22]. ILCs have been extensively studied in intestinal autoimmune diseases, especially in inflammatory bowel disease (IBD), although the exact mechanisms supporting IBD are not fully understood [23–25]. IL-17 produced by ILC3 has been shown to drive colitis, and the use of anti-IL-17 antibodies can alleviate the disease [23]. ILC3s also drive intestinal inflammation and pathology in mice with congenital colitis by secreting IL-22 and granulocyte macrophage-colony stimulating factor (GM-CSF) against CD40 [26, 27]. ILC1 is significantly increased, and ILC2 is significant decreased in patients with systemic lupus erythematosus (SLE) [28]. Therefore, increased circulating ILC1 might contribute to the pathogenesis of SLE through a type 1 immune response [28].

ANCA-associated vasculitis (AAV) consists of granulomatosis with polyangiitis (GPA), microscopic polyangiitis (MPA), and eosinophilic granulomatosis with polyangiitis (EGPA) [29]. The ILCs in GPA and MPA patients were lower in the acute phase compared with control, wheras the ILC2 and ILC3 were lower and ILC1 was higher when compared with the control or AAV patients in remission [30].

The role of ILCs in the pathogenesis of HSP has not been reported up to now. Therefore, the purpose of this paper is to investigate whether ILCs participate in the pathogenesis of HSP. The results could provide an additional hint for the understanding of the pathogenesis of HSP.

## Methods

### Study design

This was a prospective study in patients with HSP who were hospitalized at the Children’s Hospital of Soochow University from June to December 2019. This study was approved by the Ethics Committee of the Children’s Hospital Affiliated to Soochow University. Informed written consent was obtained from the legal guardians of all patients and controls.

All newly diagnosed HSP patients fulfilled the European League Against Rheumatism (EULAR) criteria for the diagnosis of HSP [31]: 1) purpura or petechiae with lower limb predominance and 2) at least one of the four following criteria: i) abdominal pain, ii) histopathological confirmation, iii) arthritis or arthralgia, and iv) renal involvement. The exclusion criteria were 1) primary sclerosing syndrome, 2) systemic sclerosis, 3) myositis, 4) scleroderma, 5) juvenile rheumatoid arthritis, 6) mucocutaneous lymph node syndrome, 7) malignant tumor, or 8) taking immunosuppressors.

If the patient had evidence of streptococcal or mycoplasma infection, antibiotic treatment was given. Conventional treatment included vitamin C and dipyridamole. Urokinase was used for HSPN. If skin damage is severe or joints, abdomen, or kidneys are involved, methylprednisone was given intravenously (1–2 mg/kg, twice a day). For patients with severe abdominal or renal symptoms, intravenous injection of large doses of immunoglobulin or plasma exchange was performed. Follow-up was performed for patients with mild hematuria, mild proteinuria, and normal renal function. A renal biopsy was performed, if necessary, to determine the next treatment plan.

Age- and sex-matched healthy patients (controls) were recruited. The same exclusion criteria were applied.

### PBMC isolation and flow cytometry for ILCs

After a patient was admitted to the hospital, heparin anticoagulation tubes were used to collect 2–3 ml of peripheral venous blood (fasting blood was not required). The patients underwent routine therapy combined with methylprednisone and were retested after 7–10 days of treatment. The peripheral blood mononuclear cells (PBMCs) were isolated using density-gradient centrifugation on Ficoll-Paque™ PLUS (#2010C1119, Cytiva, Global Life Sciences Solutions USA LLC, Marlborough, MA, USA).

Single-cell suspensions were prepared and washed three times with phosphate buffer saline (PBS). The PBMCs were stained with antibodies against CD45 (HI30, #563,204), CD117 (YB5.B8, #562,094), CD127 (HIL-7R-M21, #562,436), CRTH2 (BM16, #558,042), and a lineage cocktail (LIN) (all from Pharmingen, BD Biosciences, Franklin Lake, NJ, USA) for 15 min at room temperature in the dark (see Supplementary Table S1), with the total ILCs being defined as LIN^−^ CD45^+^ CD127^+^ CD161^+^, the ILC1 as CD117^−^ CRTH2^−^, the ILC2 as CD117^−/+^ CRTH2^+^, and the ILC3 as CD117^+^ CRTH2^−^ [32]. Data were acquired using a Gallios 3L 10c (Beckman Coulter, Brea, CA, USA) and analyzed with the FlowJo 10 software (FlowJo LLC, BD Biosciences, Franklin Lake, NJ, USA), where individual gates were established using the Fluorescence Minus One (FMO) controls.

**Fig. 1:**
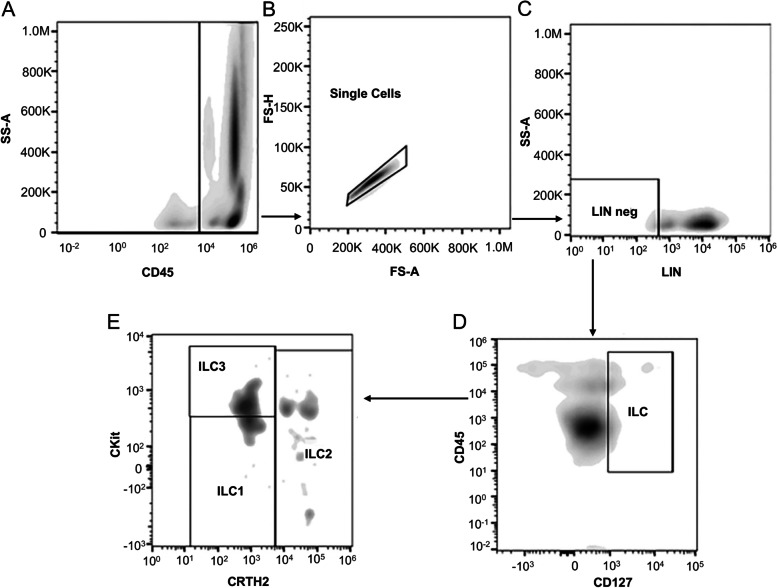
Frequency and distribution of innate lymphoid cell (ILC) subsets in the peripheral blood of patients with Henoch-Schonlein purpura (HSP). The gating strategy for total ILCs was CD45^+^ Lin^−^ CD127^+^ CD161^+^, for ILC1s was CD45^+^ Lin^−^ CD127^+^ CD161^+^ cKit^−^ CRTH2^−^, for ILC2s was CD45^+^ Lin^−^ CD127^+^ CD161^+^ cKit^−/+^ CRTH2^+^, and for ILC3s was CD45^+^ Lin^−^ CD127^+^ CD161^+^ cKit^+^ CRTH2^−^. The panels are representative of multiple independent experiments (*n* = 51)

According to the gating strategy by Sandra Bonne-Annee et al. [33], the isolated PBMCs were subjected to flow cytometry for the analysis of ILC subsets. The lineage markers (CD3, CD45, CD19, CD14, CD1a, CD94, and CD34; Supplementary Table S1) were used to exclude B lymphocytes, T lymphocytes, monocytes, dendritic cells, NK cells, and hematopoietic stem cells. Lin^−^ CD127^+^ cells gated on CD45^+^ lymphocytes were considered to be ILCs (Fig. 1A-C). To further differentiate subtypes, ILCs expressing CRTH2^+^ were defined as ILC2 (Fig. 1B, C). CD117^−^ CRTH2^−^ ILCs were designated as ILC1, and CD117^+^ CRTH2^−^ ILCs were identified as ILC3 (Fig. 1). As shown in Fig. 1B from a typical HSP patient, ILC1, ILC2, and ILC3 can be differentiated clearly.

### Data collection

Sex, age, disease involvement of clinical manifestations (purpura, abdominal, kidney, arthritis, or mixed) were collected from all patients.

### Statistical analysis

Statistical analysis was performed using SPSS 17.0 (IBM, Armonk, NY, USA). Normally distributed data (according to the Kolmogorov–Smirnov test) were presented as means ± standard deviations (SD) and analyzed using Student’s t-test or the paired t-test for intragroup comparisons; otherwise, the data were analyzed using the Mann–Whitney U-test. Categorical data are presented as n (%) and were analyzed using the chi-square test. Correlations were analyzed using the Spearman correlation coefficient. Two-tailed *P*-values < 0.05 were considered statistically significant.

## Results

### ILC subsets were altered in HSP patients

In this study, 51 patients with HSP and 22 control patients were included. There were no differences in age and sex between the two groups (Table 1). There were 6, 14, 11, 6, and 14 patients showed purpura, arthritis, abdominal symptoms, renal symptoms, and mixed symptoms.

**Table 1:** Demographic and clinical feature of the HSP patients and controls

	**HSP**	**Controls**	**P**
Sex (male/female)	22/29	6/16	0.192
Age (months)	86.3 ± 31.4	79.6 ± 27.5	0.394
Symptoms			–-
Purpura	6		
Arthritis	14		
Abdominal	11		
Renal	6		
Mixed	14		

**Fig. 2:**
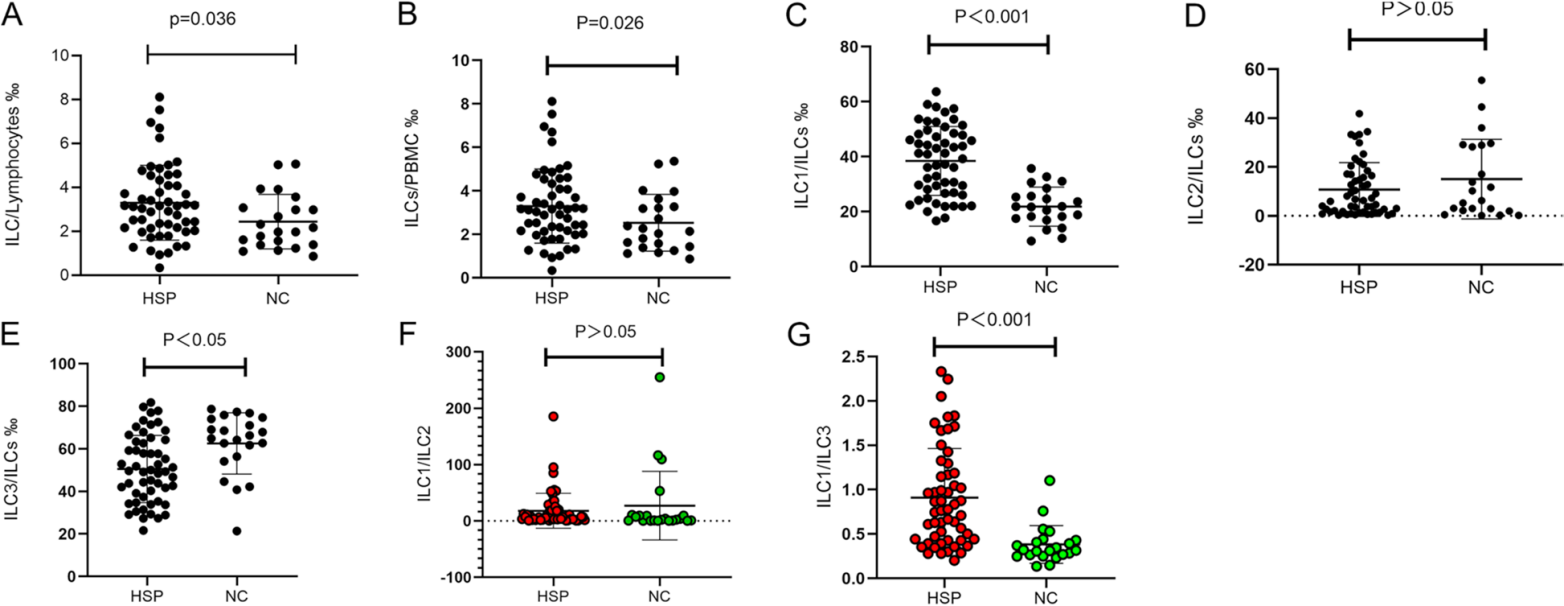
Altered frequencies of innate lymphoid cells (ILCs) in peripheral blood of patients with Henoch-Schonlein purpura (HSP). Peripheral blood mononuclear cells (PBMCs) from control and HSP patients were stained for flow cytometry. The ratios of ILC/Lymphocytes (**A**), ILC/PBMCs (**B**), ILC1/ILCs (**C**), ILC2/ILCs (**D**), ILC3/ILCs (**E**), ILC1/ILC2 (**F**), and ILC1/ILC3 (**G**) were determined in HSP (*n* = 51) and control (NC, *n* = 22) patients. NC: control patients. HSP: Henoch-Schonlein purpura

Compared with controls, patients with HSP had higher ILCs in relation to lymphocytes (*P* = 0.036), higher ILCs in relation to PBMCs (*P* = 0.026), higher ILC1s (*P* < 0.001), lower ILC3s (*P* < 0.05), and higher ILC1/ILC3 ratio (*P* < 0.001); there were no differences regarding ILC2 (*P* > 0.05) (Fig. 2).

### The ratio of ILC1/ILCs and ILC3/ILCs in HSP patients are restored after treatment

**Fig. 3:**
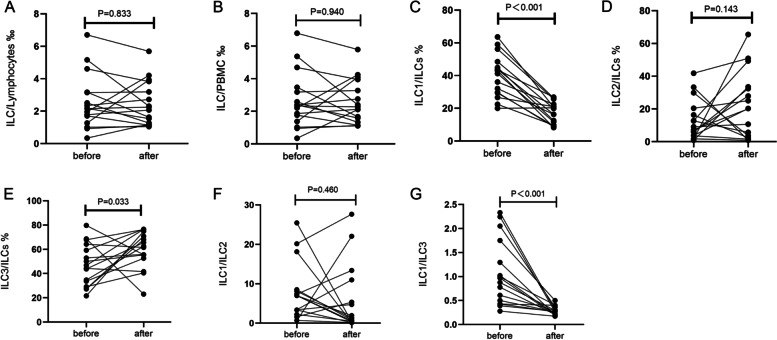
Proportions of innate lymphoid cell (ILC) subsets after treatment. Fresh blood samples from 16 patients with Henoch-Schonlein purpura (HSP) were collected on admission and prior to discharge after treatment. Peripheral blood mononuclear cells (PBMCs) of these patients were subjected to flow cytometry. The proportions of ILC/lymphocytes (**A**), ILC/PBMC (**B**), ILC1/ILCs (**C**), ILC2/ILCs (**D**), ILC3/ILCs (**E**), ILC1/ILC2 (**F**), and ILC1/ILC3 (**G**) are shown

Sixteen patients underwent routine therapy (infection control, vitamin C supplementation, loratadine, dipyridamole), combined with methylprednisolone merely during acute abdominal pain and joint pain periods, for 7–10 days. There were no changes in ILCs/Lymphocytes and ILCs/PBMC before and after treatment (*P* = 0.833 and *P* = 0.940, respectively) (Fig. 3 and Supplementary Table S2). As shown in Fig. 3 and Supplementary Table S2, ILC1s were significantly decreased (*P* < 0.001), and ILC3s were increased (*P* = 0.033), while there were no significant changes in ILC2(*P* = 0.143). In addition, ILC1/ILC3 was significantly decreased after treatment (*P* < 0.001), while there were no differences in the ratio of ILC1/ILC2 before and after treatment (*P* = 0.460).

### ILC subsets in HSP with different symptoms

**Fig. 4:**
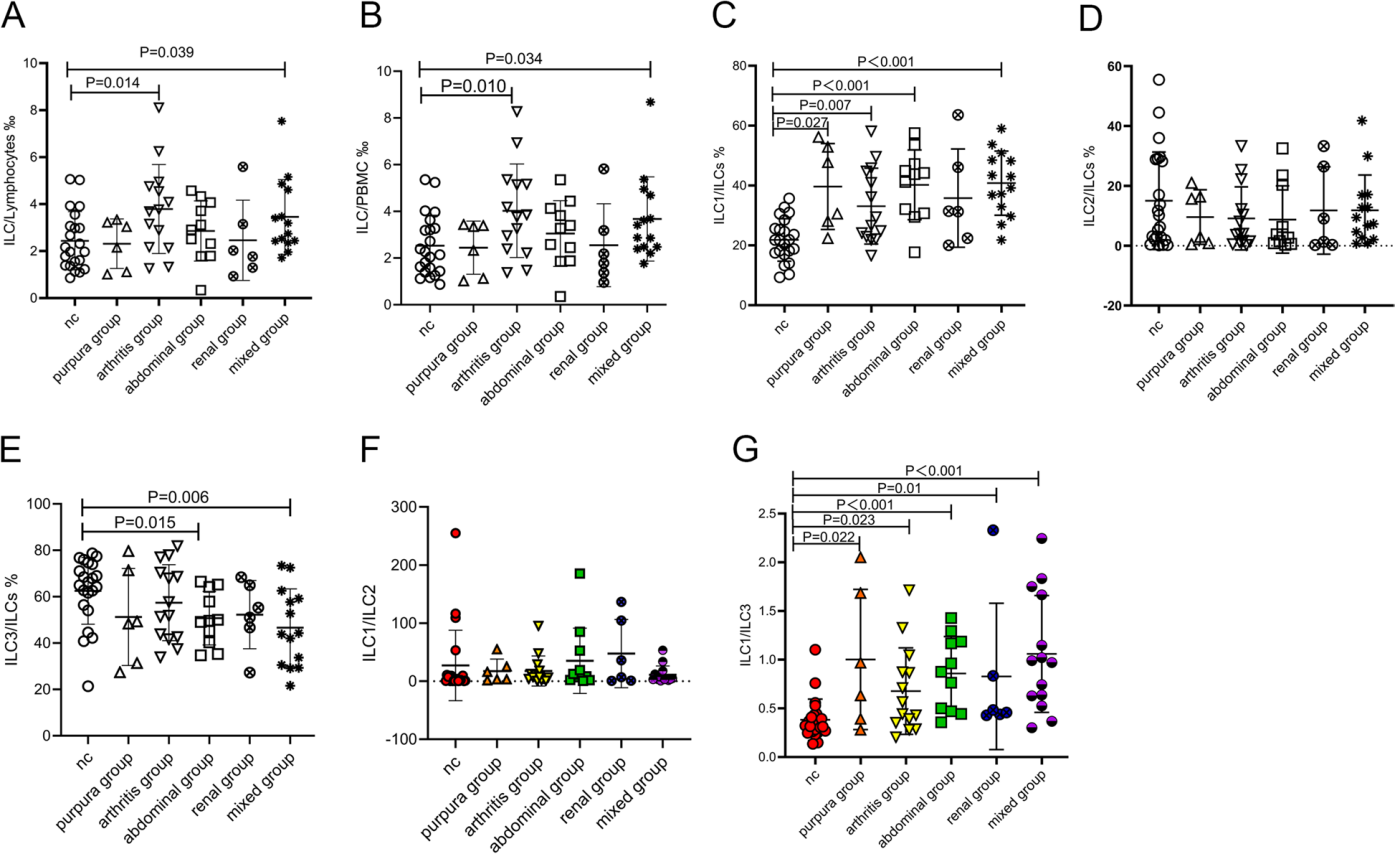
Alteration of innate lymphoid cell (ILC) subsets in patients with Henoch-Schonlein purpura (HSP). Peripheral blood mononuclear cells (PBMCs) were collected from HSP and control patients. The HSP patients (*n* = 51) were assessed by the purpura group (*n* = 6), arthritis group (*n* = 14), abdominal group (*n* = 11), renal group (*n* = 6), and mixed group (*n* = 14). The proportions of ILC/lymphocytes (**A**), ILC/PBMC (**B**), ILC1/ILCs (**C**), ILC2/ILCs (**D**), ILC3/ILCs (**E**), ILC1/ILC2 (**F**), and ILC1/ILC3 (**G**) of each subset of HSP group and the control group are presented. NC: control patients. HSP, Henoch-Schonlein purpura

Purpura, was the most typical clinical manifestation of the enrolled HSP patients, whereas there were other representative manifestations as well. According to their clinical manifestations, the patients of HSP were divided into five subgroups: purpura group (patients presenting only with typical skin Purpura, refer to the domestic and foreign classification criteria [34]), arthritis group (patients with skin Purpura and acute onset arthralgia or arthritis), abdominal group (patients with skin Purpura and acute onset diffuse abdominal colicky pain), renal group (patients with skin purpura and nephritis symptoms, such as hematuria or proteinuria, or IgA deposits in the kidney indicated by renal biopsy), and mixed symptoms (possessing more than two symptoms as stated above). Compared with the controls, the ratios of ILCs/lymphocytes were higher in patients in the arthritis and mixed groups (*P* = 0.014 and *P* = 0.039 respectively), and the ILCs/PBMC ratios were higher in the same two groups (*P* = 0.010 and *P* = 0.034). ILC1 were elevated in the purpura, arthritis, abdominal, and mixed groups (*P* = 0.027, *P* = 0.007, *P* < 0.001, and *P* < 0.001, respectively). ILC3s were lower in the abdominal and mixed groups (*P* = 0.015 and *P* = 0.006). The ILC1/ILC3 ratios in all subgroups of HSP were higher than that of the control group (all *P* < 0.05) (Fig. 4).

### Immunocytes are correlated with ILC1/ILCs

**Fig. 5:**
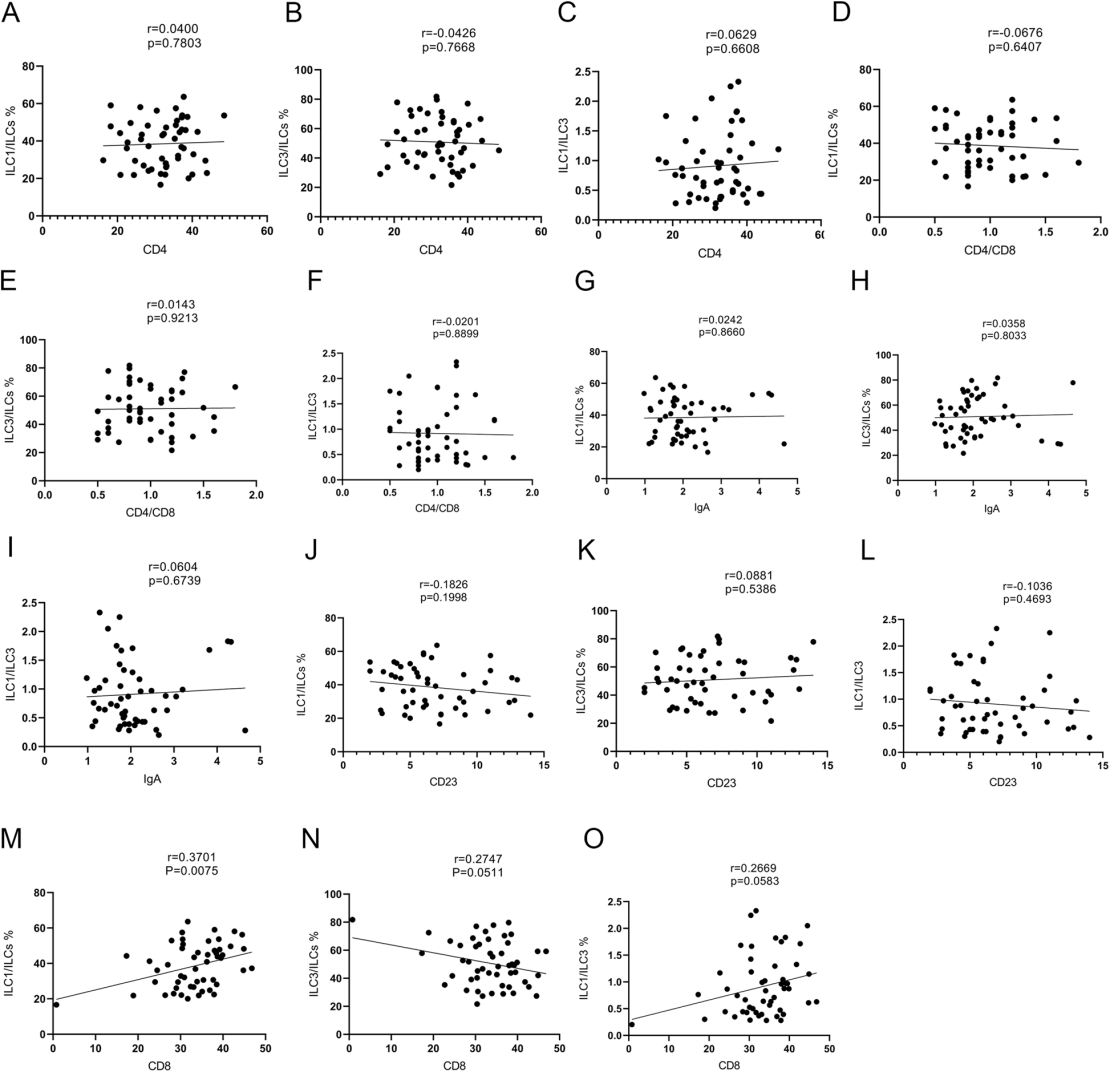
Correlation between innate lymphoid cell (ILC) subsets and lymphocyte subsets. CD8 + was positively correlated with ILC1/ILCs (M). The correlation between the levels of CD4 + (A-C), CD4 + /CD8 + (D-F), IgA (G-I), CD23 + (J-L), CD8 + (M–O) in patients with Henoch-Schonlein purpura (HSP) and ILC subsets are shown. Each point represents an individual subject

ILC1/ILCs were positively correlated with CD3^+^ CD8^+^ T lymphocytes (*r* = 0.3701, *P* = 0.0075) (Fig. 5). We did not find correlations between other ILC subsets and levels of CD4 + (A-C), CD4 + /CD8 + (D-F), IgA (G-I), CD23 + (J-L), CD8 + (N–O) and in patients with HSP (Fig. 5).

## Discussion

ILC dysfunction is involved in numerous immune diseases, but this has not been demonstrated in HSP. This study aimed to investigate whether ILC dysfunction or imbalance participate in the pathogenesis of HSP. The results suggest that higher circulating ILC1s and lower circulating ILC3s appear to be involved in the pathogenesis of HSP.

ILCs are a heterogeneous population of non-B non-T lymphocytes that can provide an immediate immune response that prevents pathogen invasion and affects the subsequent adaptive immune responses [11]. Few ILCs are detected in peripheral blood (accounting for 0.1–0.5% of lymphocytes) and cord blood [32]. ILCs are mainly enriched in barrier surfaces, NCR^+^ ILCs account for approximately 5% and 2% of the total lymphocytes in the human and mouse small intestine, respectively [35]. ILCs have also been found in human gingivae. Approximately 10%-15% of total CD45^+^ cells are ILCs, most of which are IFN-γ secreting ILC1s [36]. ILCs play an important role in maintaining barrier homeostasis, blocking bacterial and viral infections, expelling parasites, tissue repair, and tumor immunity. ILCs are dysregulated in multiple human diseases [37], including allergic inflammation and asthma [17–22], and IBD [23–25]. The main ILC population involved in airway inflammation appears to be ILC2 [17–22], while ILC3 appears to be the population the most involved in IBD [23–25]. HSP is an immune disease with injury to the skin and intestinal mucosa. Cytokines secreted from damaged epithelial cells may stimulate ILC proliferation [9, 10]. In this study, ILC1s were higher and ILC3s were lower than in controls, without significant changes in ILC2. This suggests that the different ILC populations play different roles in different immune conditions. The course of HSP is generally about 1–3 months, and a few can last several months or more than a year. If it is accompanied by renal disease, such as chronic kidney disease or even chronic renal insufficiency, then it will present the characteristics of chronic disease. Asthma and inflammatory bowel disease are also chronic conditions. They are both immune diseases, but the conclusions are different, which may be related to different immune states. This is worth exploring in the future.

ILC1 can block viral, intracellular bacterial, and fungal infection through secreting IFN-γ, but ILC1 could also promote COPD, IBD, and cancer in response to different stimuli [23, 24, 38–40]. The absence of ILC1 in T-bet^−/−^ mice increased the sensitivity to enteric infections [41]. Several groups demonstrated that the percentage of ILC1 is increased in the intestines of patients with Crohn’s disease [42, 43] and affect the disease severity through excessive cytokine production. The proportion of ILC1 is increased obviously with significant decreases of ILC2 in SLE patients, while, in patients with moderate to severe activity, ILC3 also decreases visibly [28]. In this study, ILC1 increased on the early onset with ILC3 decreased, which was consistent with the previous reports, indicating that ILC1 may be involved in the pathogenesis of HSP. The percentage of ILCs and ILC3 in peripheral blood mononuclear cells (PBMC) was reduced in patients with lupus nephritis [44], whereas in our study, compared with controls, there were no difference in ILCs/lymphocyte ratio or ILCs/PBMC ratio. We speculate that it may be related to the insufficient number of patients and the relatively mild nature of the disease.In our study, there was no difference in ILC2, which may be due to the stromal adventitial cells secreting IL-33 promoting the stability of ILC2 in blood circulation and renal tissue.

The development and function of ILC3s require the transcription factor RORγt and the production of IL-17A and/or IL-22 [45]. IL-22 produced by ILC3s induces epithelial cells to express anti-bacterial peptides such as Reg3g and Reg3b to block microbiota and pathogens as well as stabilize the epithelial barrier. ILC3s play a critical role in the injury repair of epithelial cells [46]. ILC3 could limit T follicular helper (TFH) cells responses and B cell class switching through antigen presentation within the interfollicular regions of the intestinal draining lymph nodes [47]. In this study, ILC3s were lower than in controls and were restored after treatment. The ratio of ILC1/ILC3 was also reduced significantly after treatment. Previous data indicate that ILCs have the feature of plasticity [14, 15]. Therefore, it could be hypothesized that ILC3 might play a role in resistance to foreign pathogens by transforming into ILC1 in the acute phase of HSP, and ILC1 might be converted into ILC3 in the convalescence of the disease. This will have to be verified.

Emerging evidence indicates that there is a complex regulatory relationship between innate immunity and adaptive immunity [48]. ILC3 in the intestinal tract and lymphoid tissues act on stromal cells and secrete lymphotoxin (LT)α and LTβ to help B cell class switching and IgA production indirectly [49]. There were no correlations between IgA and ILC1, ILC3, and ILC1/ILC3 in this study. It was speculated that ILCs mainly exist in the mucosal lamina propria and less in peripheral blood, so it had less effect on serum IgA. The effect of ILCs on IgA can be further elucidated by expounding the relationship between ILCs in the mucosa lamina propria and secretory IgA. This will have to be examined in future studies.

ILCs subsets closely mirror the transcriptional and functional biology of both cytotoxic CD8^+^ T cells and CD4^+^ T helper (Th) cells. IFN-γ-producing ILC1s modulate T cell responses, especially Th1 cell responses. Macrophages can also participate in the activation of ILC3s, so ILCs are the bridge between innate immunity and adaptive immunity [48]. There was a positive correlation between ILC1 and CD3^+^ CD8^+^ cells in this study, while there were no correlations between CD3^+^ CD4^+^ cells and different subtypes of ILCs in the peripheral blood, but mucosal ILCs were not examined. This might also explain why ILCs were not associated with CD19^+^ CD23^+^ cells. More experiments are needed to indicate whether ILCs are correlated with IgA^+^ B lymphocytes.

This study has limitations. The patients were from a single center, and since HSP is an immune disease, the sample size was small. In addition, the patients were not followed for recurrence. In this study, there were differences in ILCs, ILC1s, ILC2s, and ILC3s in along with the different symptoms of HSP compared with the control group. It was speculated that ILCs might be related to the severity of HSP or may be due to different predisposing factors of HSP. Thus, it is necessary to further study the role of ILCs in HSP by expanding the sample size and extending the follow-up time.

## Conclusions

We found that peripheral blood ILC1s were higher and ILC3s were lower in HSP and that the ILC1/ILC3 ratio decreased significantly after treatment, indicating that ILC1 and ILC3 might be involved in the pathogenesis of HSP and might be correlated with the severity of the disease. Those results provide an additional understanding of the pathogenesis of HSP. Future studies should examine mucosal ILCs as well as IgA-secreting cells.

## Supplementary Information

**Additional file 1.**

**Additional file 2.**

## Data Availability

All data generated or analyzed during this study are included in this published article.

## Abbreviations

ILC: Innate lymphoid cell
HSP: Henoch-Schonlein purpura
NK: Natural killer
LTi: Lymphoid-tissue inducer
IBD: Inflammatory bowel disease
GM-CSF: Granulocyte macrophage-colony stimulating factor
SLE: Systemic lupus erythematosus
EULAR: European League Against Rheumatism
PBMCs: Peripheral blood mononuclear cells
PBS: Phosphate buffer saline
LIN: Lineage cocktail
FMO: Fluorescence Minus One
SD: Standard deviations
TFH: T follicular helper
Th: T helper

## References

[1] McCarthy HJ, Tizard EJ (2010). Clinical practice: diagnosis and management of Henoch-Schonlein purpura. Eur J Pediatr.

[2] Hetland LE, Susrud KS, Lindahl KH, Bygum A (2017). Henoch-Schonlein purpura: a literature review. Acta Derm Venereol.

[3] Ozen S, Sag E. Childhood vasculitis. Rheumatology (Oxford). 2020;59(Supplement_3):iii95–100. 10.1093/rheumatology/kez599. 10.1093/rheumatology/kez599 32348513

[4] Jelusic M, Sestan M, Cimaz R, Ozen S (2019). Different histological classifications for Henoch-Schonlein purpura nephritis: which one should be used?. Pediatr Rheumatol Online J.

[5] KDIGO Clinical Practice Guideline for Glomerulonephritis. International Society of Nephrology. 2020;2 Supplement 2. https://kdigo.org/wp-content/uploads/2017/02/KDIGO-2012-GN-Guideline-English.pdf.

[6] Reamy BV, Williams PM, Lindsay TJ (2009). Henoch-Schonlein purpura. Am Fam Physician.

[7] Heineke MH, Ballering AV, Jamin A, Ben Mkaddem S, Monteiro RC, Van Egmond M (2017). New insights in the pathogenesis of immunoglobulin a vasculitis (Henoch-Schonlein purpura). Autoimmun Rev.

[8] Louie CY, Gomez AJ, Sibley RK, Bass D, Longacre TA (2018). Histologic features of gastrointestinal tract biopsies in IgA vasculitis (Henoch-Schonlein Purpura). Am J Surg Pathol.

[9] Gao X, Miao R, Tao Y, Chen X, Wan C, Jia R (2018). Effect of montmorillonite powder on intestinal mucosal barrier in children with abdominal Henoch-Schonlein purpura: A randomized controlled study. Medicine (Baltimore).

[10] Ozen S, Marks SD, Brogan P, Groot N, de Graeff N, Avcin T (2019). European consensus-based recommendations for diagnosis and treatment of immunoglobulin a vasculitis-the SHARE initiative. Rheumatology (Oxford).

[11] Klose CS, Artis D (2016). Innate lymphoid cells as regulators of immunity, inflammation and tissue homeostasis. Nat Immunol.

[12] Eberl G, Colonna M, Di Santo JP, McKenzie AN (2015). Innate lymphoid cells. Innate lymphoid cells: a new paradigm in immunology. Science.

[13] Bar-Ephraim YE, Mebius RE (2016). Innate lymphoid cells in secondary lymphoid organs. Immunol Rev.

[14] DuPage M, Bluestone JA (2016). Harnessing the plasticity of CD4(+) T cells to treat immune-mediated disease. Nat Rev Immunol.

[15] Johnson JL, Vahedi G (2018). Do memory CD4 T cells keep their cell-type programming: plasticity versus fate commitment? epigenome: a dynamic vehicle for transmitting and recording cytokine signaling. Cold Spring Harb Perspect Biol..

[16] Ebbo M, Crinier A, Vely F, Vivier E (2017). Innate lymphoid cells: major players in inflammatory diseases. Nat Rev Immunol.

[17] Kubo M (2017). Innate and adaptive type 2 immunity in lung allergic inflammation. Immunol Rev.

[18] Peebles RS, Aronica MA (2019). Proinflammatory pathways in the pathogenesis of asthma. Clin Chest Med.

[19] Cai T, Qiu J, Ji Y, Li W, Ding Z, Suo C (2019). IL-17-producing ST2(+) group 2 innate lymphoid cells play a pathogenic role in lung inflammation. J Allergy Clin Immunol.

[20] Wallrapp A, Riesenfeld SJ, Burkett PR, Abdulnour RE, Nyman J, Dionne D (2017). The neuropeptide NMU amplifies ILC2-driven allergic lung inflammation. Nature.

[21] Huang Y, Mao K, Chen X, Sun MA (2018). S1P-dependent interorgan trafficking of group 2 innate lymphoid cells supports host defense. Science.

[22] Van Maele L, Carnoy C, Cayet D, Ivanov S, Porte R, Deruy E (2014). Activation of Type 3 innate lymphoid cells and interleukin 22 secretion in the lungs during Streptococcus pneumoniae infection. J Infect Dis.

[23] Zeng B, Shi S, Ashworth G, Dong C, Liu J, Xing F (2019). ILC3 function as a double-edged sword in inflammatory bowel diseases. Cell Death Dis.

[24] Geremia A, Arancibia-Carcamo CV (2017). Innate Lymphoid cells in Intestinal Inflammation. Front Immunol.

[25] Geremia A, Biancheri P, Allan P, Corazza GR, Di Sabatino A (2014). Innate and adaptive immunity in inflammatory bowel disease. Autoimmun Rev.

[26] Pearson C, Thornton EE, McKenzie B, Schaupp AL, Huskens N, Griseri T (2016). ILC3 GM-CSF production and mobilisation orchestrate acute intestinal inflammation. Elife.

[27] Hepworth MR, Fung TC, Masur SH, Kelsen JR, McConnell FM, Dubrot J (2015). Immune tolerance. Group 3 innate lymphoid cells mediate intestinal selection of commensal bacteria-specific CD4(+) T cells. Science.

[28] Guo C, Zhou M, Zhao S, Huang Y, Wang S, Fu R (2019). Innate lymphoid cell disturbance with increase in ILC1 in systemic lupus erythematosus. Clin Immunol.

[29] Jennette JC (2013). Overview of the 2012 revised International Chapel Hill Consensus Conference nomenclature of vasculitides. Clin Exp Nephrol.

[30] Braudeau C, Amouriaux K, Néel A, Herbreteau G, Salabert N, Rimbert M (2016). Persistent deficiency of circulating mucosal-associated invariant T (MAIT) cells in ANCA-associated vasculitis. J Autoimmun.

[31] Ozen S, Pistorio A, Iusan SM, Bakkaloglu S, Herlin T, Brik R (2010). EULAR/PRINTO/PRES criteria for Henoch-Schonlein purpura, childhood polyarteritis nodosa, childhood Wegener granulomatosis and childhood Takayasu arteritis: Ankara 2008. part II: final classification criteria. Ann Rheum Dis.

[32] Bianca Bennstein S, Riccarda Manser A, Weinhold S, Scherenschlich N, Uhrberg M (2019). OMIP-055: characterization of human innate lymphoid cells from neonatal and peripheral blood. Cytometry A.

[33] Bonne-Annee S, Bush MC, Nutman TB (2019). differential modulation of human Innate Lymphoid Cell (ILC) subsets by IL-10 and TGF-beta. Sci Rep.

[34] Cui J, Huang LY, Guo J, Wu CR, Zhang B (2019). Diagnosis and treatment of adult mixed-type Henoch-Schonlein purpura. Cent Eur J Immunol.

[35] Buela KA, Omenetti S, Pizarro TT (2015). Cross-talk between type 3 innate lymphoid cells and the gut microbiota in inflammatory bowel disease. Curr Opin Gastroenterol.

[36] Dutzan N, Konkel JE, Greenwell-Wild T, Moutsopoulos NM (2016). Characterization of the human immune cell network at the gingival barrier. Mucosal Immunol.

[37] Wu J, Lv X (2019). Critical roles of balanced innate lymphoid cell subsets in intestinal homeostasis, chronic inflammation, and cancer. J Immunol Res.

[38] Borger JG, Lau M, Hibbs ML (2019). The Influence of innate lymphoid cells and unconventional T Cells in chronic inflammatory lung disease. Front Immunol.

[39] Panda SK, Colonna M (2019). Innate lymphoid cells in mucosal immunity. Front Immunol.

[40] Forkel M, van Tol S, Hoog C, Michaelsson J, Almer S, Mjosberg J. Distinct alterations in the composition of mucosal innate lymphoid cells in newly diagnosed and established Crohn’s disease and ulcerative colitis. J Crohns Colitis. 2019;13(1):67–78. 10.1093/ecco-jcc/jjy119. 10.1093/ecco-jcc/jjy119 30496425

[41] Klose CS, Kiss EA, Schwierzeck V, Ebert K, Hoyler T, d’Hargues Y, et al. A T-bet gradient controls the fate and function of CCR6-RORgammat+ innate lymphoid cells. Nature. 2013;494(7436):261–5. 10.1038/nature11813. 10.1038/nature11813 23334414

[42] Geremia A, Arancibia-Carcamo CV, Fleming MP, Rust N, Singh B, Mortensen NJ (2011). IL-23-responsive innate lymphoid cells are increased in inflammatory bowel disease. J Exp Med.

[43] Fuchs A, Vermi W, Lee JS, Lonardi S, Gilfillan S, Newberry RD (2013). Intraepithelial type 1 innate lymphoid cells are a unique subset of IL-12- and IL-15-responsive IFN-gamma-producing cells. Immunity.

[44] Ryu S, Lee EY, Kim DK, Kim YS, Chung DH, Kim JH (2020). Reduction of circulating innate lymphoid cell progenitors results in impaired cytokine production by innate lymphoid cells in patients with lupus nephritis. Arthritis Res Ther.

[45] Vivier E, Artis D, Colonna M, Diefenbach A, Di Santo JP, Eberl G (2018). Innate lymphoid cells: 10 years on. Cell.

[46] Melo-Gonzalez F, Hepworth MR (2017). Functional and phenotypic heterogeneity of group 3 innate lymphoid cells. Immunology.

[47] Melo-Gonzalez F, Kammoun H, Evren E (2019). Antigen-presenting ILC3 regulate T cell-dependent IgA responses to colonic mucosal bacteria. J Exp Med.

[48] Sonnenberg GF, Hepworth MR (2019). Functional interactions between innate lymphoid cells and adaptive immunity. Nat Rev Immunol.

[49] Li S, Bostick JW, Zhou L (2017). Regulation of innate lymphoid cells by Aryl Hydrocarbon Receptor. Front Immunol.

