# Supplementary material for: Increased circulating innate lymphoid cell (ILC)1 and decreased circulating ILC3 are involved in the pathogenesis of Henoch-Schonlein purpura
Source: BMC Pediatr. 2022 Apr 12;22:201. doi: 10.1186/s12887-022-03262-w (PMC9003988; doi:10.1186/s12887-022-03262-w)
Supplement: Supplementary file 1 — Additional file 1. [file 12887_2022_3262_MOESM1_ESM.docx]

**Supplementary Table S1** ILC staining panel for flow cytometry

|  | **Target** | **Color** | **Clones** | **Source** | **Catalog #** |
| --- | --- | --- | --- | --- | --- |
| Lineage markers | CD3 | FITC | UCHT1 | BD | 561806 |
|  | CD1a | FITC | HI149 | BD | 560945 |
|  | CD14 | FITC | M5E2 | BD | 561712 |
|  | CD19 | FITC | HIB19 | BD | 560994 |
|  | CD94 | FITC | HP-3D9 | BD | 555888 |
|  | CD34 | FITC | 581 | BD | 343503 |
|  | CD45 | BV510 | HI30 | BD | 563204 |
| ILC subset markers | CD127 | BV421 | HIL-7R-M21 | BD | 562436 |
|  | CD161 | PE | DX12 | BD | 556081 |
|  | CRTH2 (CD294) | Alexa 647 | BM16 | BD | 558042 |
|  | CD117 (c-kit) | PerCP-Cy5.5 | YB5.B8 | BD | 562094 |

ILC: innate lymphoid cell
