# Supplementary material for: Increased circulating innate lymphoid cell (ILC)1 and decreased circulating ILC3 are involved in the pathogenesis of Henoch-Schonlein purpura
Source: BMC Pediatr. 2022 Apr 12;22:201. doi: 10.1186/s12887-022-03262-w (PMC9003988; doi:10.1186/s12887-022-03262-w)
Supplement: Supplementary file 2 — Additional file 2. [file 12887_2022_3262_MOESM2_ESM.docx]

**Supplementary Table S2** ILC and lymphocytes in HSP patients before and after treatments

| **Parameter** | **Before treatment** | **After treatment** | ***P*** |
| --- | --- | --- | --- |
| ILCs/lymphocytes (‰) | 2.56±1.68 | 2.45±1.38 | 0.833 |
| ILCs/PBMC (‰) | 2.67±1.71 | 2.56±1.37 | 0.940 |
| ILC1/ILCs (%) | 39.99±12.91 | 17.23±6.55 | <0.001 |
| ILC2/ILCs (%) | 12.97±12.42 | 21.91±20.31 | 0.143 |
| ILC3/ILCs (%) | 46.94±17.41 | 59.82±15.17 | 0.033 |
| ILC1/ILC2 | 46.94±17.41 | 5.65±8.56 | 0.460 |
| ILC1/ILC3 | 1.06±0.68 | 0.29±0.09 | <0.001 |

ILC, innate lymphoid cell; PBMC, peripheral blood mononuclear cells
